# A Clot Waveform Analysis of Thrombin Time Using a Small Amount of Thrombin Is Useful for Evaluating the Clotting Activity of Plasma Independent of the Presence of Emicizumab

**DOI:** 10.3390/jcm11206142

**Published:** 2022-10-18

**Authors:** Hideo Wada, Katsuya Shiraki, Takeshi Matsumoto, Kei Suzuki, Yoshiki Yamashita, Isao Tawara, Hideto Shimpo, Motomu Shimaoka

**Affiliations:** 1 Department of General and Laboratory Medicine, Mie Prefectural General Medical Center, Yokkaichi 510-0885, Japan; 2 Department of Transfusion Medicine and Cell Therapy, Mie University Hospital, Tsu 514-8507, Japan; 3 Emergency Critical Care Center, Mie University Graduate School of Medicine, Tsu 514-8507, Japan; 4 Department of Hematology and Oncology, Mie University Graduate School of Medicine, Tsu 514-8507, Japan; 5 Mie Prefectural General Medical Center, Yokkaichi 510-0885, Japan; 6 Department of Molecular Pathobiology and Cell Adhesion Biology, Mie University Graduate School of Medicine, Tsu 514-8507, Japan

**Keywords:** CWA-APTT, CWA-TT, emicizumab, FVIII activity, monitoring, thrombin burst

## Abstract

Objective: Although emicizumab is a bispecific, monoclonal antibody that has led to a significant improvement of treatment for hemophilia A patients with inhibitors, the routine monitoring of patients treated with emicizumab is difficult. Thrombin time (TT) reflects thrombin burst, which mainly depends on activation of factor V (FV) and FVIII. Methods: We, therefore, developed a method for evaluating clotting activity independent of the presence of emicizumab. Normal plasma (NP) or FVIII-deficient plasma (FVIIIDP) with and without emicizumab was measured using clot waveform analysis (CWA)-activated partial thromboplastin time (APTT) and TT. Results: Emicizumab caused clot formation in FVIIIDP using the CWA-APTT; however, the coagulation peaks of plasma with and without emicizumab measured by the CWA-TT did not differ to a statistically significant extent. Regarding the mixing tests with NP and FVIIIDP, CWA-APTT showed large differences between each mixing test in plasma with and without emicizumab, whereas the CWA-TT showed similar patterns in mixing plasma with and without emicizumab. Regarding the standard curve of FVIII activity, the CWA-APTT showed an FVIII-concentration-dependent increase; however, the values with each concentration of FVIII differed between samples with and without emicizumab, whereas CWA-TT showed FVIII-concentration-dependent fluctuations independent of the presence of emicizumab, and the values with each concentration of FVIII were similar in samples with and without emicizumab. Conclusions: As CWA-TT using a small amount of thrombin (0.5 IU/mL) can reflect thrombin burst and be useful for evaluating FVIII activity, independent of the presence of emicizumab, it is useful for monitoring clotting activity in patients with an anti-FVIII inhibitor treated with emicizumab.

## 1. Introduction

The mortality in patients with hemophilia A has decreased because of advances in the treatment of bleeding [1]. Although prophylactic treatment with coagulation factor VIII (FVIII) concentrate is preferred to prevent bleeding and joint damage in children with severe hemophilia [2,3], there are still several risk factors for the treatment of hemophilia A [4]. One of the most important risk factors for the treatment of hemophilia A is posed by inhibitors of FVIII [5]. Recently, emicizumab, which is a bispecific, monoclonal antibody that bridges activated factor IX and factor X to replace the function of missing activated factor VIII, has been developed and has led to the significant improvement in treatment of hemophilia A patients with inhibitors [6,7]. Although the activated partial thromboplastin time (APTT) is a clotting time assay that is useful in screening for hemophilia [8] and the presence of inhibitors [9,10], and for monitoring unfractionated heparin treatment [11], this assay is not suitable for patients treated with emicizumab, because the APTT in patients treated with emicizumab is significantly shortened and is not able to measure FVIII activity in these patients [12]. The effects of emicizumab on several assays have been reported [13].

An optical automatic coagulation analyzer has been able to demonstrate the clot reaction curve of APTT [14,15], diluted prothrombin time (PT) [16] and thrombin time (TT) [17]. Such an analysis of the coagulation curve is called a clotting waveform analysis (CWA) [18]. Furthermore, a new FVIII assay using a small amount of tissue-factor-induced FIX activation (sTF/FIXa) has also been reported to be useful for measuring FVIII levels, which are activated via the extrinsic pathway [19]. In addition, sTF/FIXa may reflect thrombin burst, which mainly depends on the activation of FXI, FVIII and FV [20,21]. CWA-TT has also been reported to be useful for evaluating thrombin burst [17]. Although measurement of FVIII activity in patients treated with emicizumab using anti-idiotype, monoclonal antibodies is possible [22], this method is not easy to perform.

As emicizumab will likely be frequently used for patients with acquired hemophilia A in the future, the correct measurement of FVIII activity in patients receiving emicizumab will be important for deciding whether or not to discontinue the drug’s administration. In this study, we show that CWA-TT using a low concentration of thrombin is a useful method for measuring FVIII activity independent of the presence of emicizumab.

## 2. Materials and Methods

The CWA-TT was measured using 0.5 IU thrombin (500 units of thrombin, Mochida Pharmaceutical Co., Ltd., Tokyo, Japan) with an ACL-TOP^®^ system (Instrumentation Laboratory, Bedford, MA, USA) [17]. Three types of curves are shown on this system monitor [21]. One shows the changes in absorbance observed while measuring the TT, corresponding to the fibrin formation curve (FFC). The second is the first derivative peak of absorbance (1st DP), corresponding to the coagulation velocity. The third is the second derivative peak of absorbance (2nd DP), corresponding to coagulation acceleration. FVIII-deficient plasma (Instrumentation Laboratory) was used as clotting-factor-deficient plasma, and calibration plasma (Instrumentation Laboratory) was used as normal plasma. Emicizumab was kindly supplied by Chugai Pharmaceutical Co., Ltd. (Tokyo, Japan).

The CWA-APTT was measured using a HemosIL APTT-SP (Instrumentation Laboratory), as previously reported [23]. The sTF/FIXa assay was performed using C.K.Prest (Diagnostica Stago S.A.S., Asnières-sur-Seine, France), 10 IU/mL of FIX (Nonacog Alfa; Pfizer, Tokyo, Japan) and 2000-fold-diluted HemosIL RecombiPlasTin 2G (Instrumentation Laboratory) with an ACL-TOP^®^ system [19].

A mixing test with normal and FVIII-deficient plasma or plasma from a hemophilia A patient with an inhibitor treated with emicizumab was performed using CWA-APTT or CWA-TT. The FVIII activity was measured by the one-step clotting method using APTT-SP reagents (CWA-APTT) or 0.5 IU thrombin (CWA-TT) in an ACL-TOP system [19]. All assays were performed more than three times to confirm the reproducibility. Informed consent was obtained from the hemophilia A patient with an inhibitor treated with emicizumab.

### Statistical Analyses

Data are expressed as the mean ± standard deviation in Table 1 and the median with 25th–75th percentile in the figures. Differences between samples with and without emicizumab were examined for significance using Student’s *t*-test. *p*-values ≤ 0.05 were considered to indicate statistical significance. All statistical analyses were performed using the Stat Flex software program (version 6; Artec Co., Ltd., Osaka, Japan).

## 3. Results

**Figure 1:**
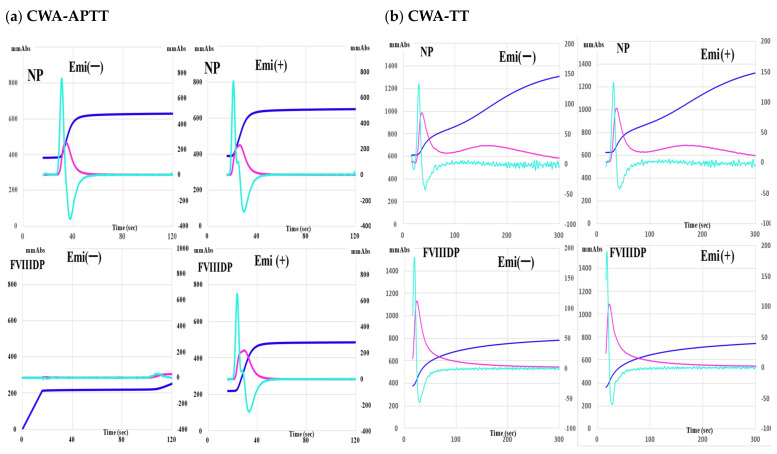
Effects of emicizumab (0.15 mg/mL) on the clot waveform analysis (CWA). (**a**) Activated partial thromboplastin time (APTT). (**b**) thrombin time (TT); upper, normal plasma (NP); lower, FVIII-deficient plasma (FVIIID); Emi, emicizumab; navy-blue, fibrin formation curve; pink curve, 1st derivative curve; light-blue curve, 2nd derivative curve. All assays were performed more than three times to confirm the reproducibility.

Emicizumab (0.15 mg/mL) caused clot formation in FVIII-deficient plasma, causing the peak of CWA-APTT to appear, whereas it shortened the peak time of the CWA-APTT in normal plasma but did not increase the peak height of the CWA-APTT (Figure 1a). Regarding the sTF/FIXa assay, emicizumab slightly decreased the peak heights and enlarged the peak width in both normal and FVIII-deficient plasma (Supplementary Figure S1). The peak height of the FF curve was significantly higher in normal plasma than in FVIII-deficient plasma. Regarding the CWA-TT using 0.5 IU/mL of thrombin, the peak times and heights of CWA-TT in both normal and FVIII-deficient plasma were similar between samples with and without emicizumab, whereas the peak heights of the FF curve with and without emicizumab were markedly higher (*p* < 0.001) in normal plasma (1366 ± 67 mm absorbance and 1380 ±56 mm absorbance, respectively) than in FVIII-deficient plasma (771 ± 54 mm absorbance and 758 ± 37 mm absorbance, respectively) (Figure 1b).

**Figure 2:**
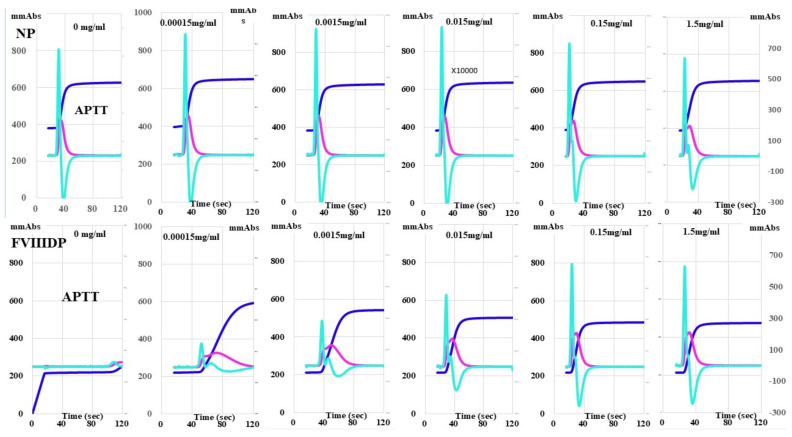
Effects of emicizumab on the clot waveform analysis (CWA)-activated partial thromboplastin time (APTT). Upper, NP (normal plasma); FVIIID, FVIII-deficient plasma; number mg/mL, emicizumab concentration; navy-blue, fibrin formation curve; pink curve, 1st derivative curve; light-blue curve, 2nd derivative curve. All assays were performed more than three times to confirm the reproducibility.

Although the peak time of the CWA-APTT in normal plasma was gradually shortened according to the concentration of emicizumab, there were no marked differences in peak heights among various concentrations of emicizumab (Figure 2). In FVIII-deficient plasma, emicizumab (0.00015 mg/mL) generated a small clot waveform, and it gradually increased the peak heights and shortened the peak times. The CWA-TT of normal and FVIII-deficient plasma did not differ among the various concentrations of emicizumab (Supplementary Figure S2).

**Figure 3:**
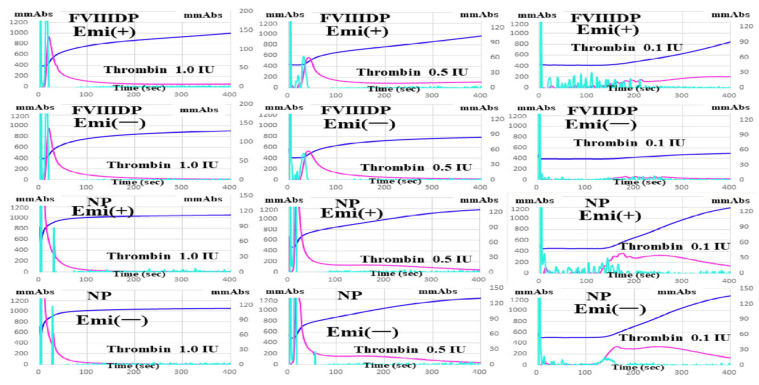
Clotting activation by thrombin using clot waveform analysis of thrombin time. NP, normal plasma; FVIIIDP, FVIII-deficient plasma; Emi, emicizumab. navy-blue, fibrin formation curve; pink curve, 1st derivative curve; light-blue curve, 2nd derivative curve. All assays were performed more than three times to confirm the reproducibility.

Under conditions of ≥0.5 IU thrombin, CWA-TT showed a similar pattern between plasma with and without emicizumab. However, under conditions of <0.5 IU, clot formation by thrombin was stronger in normal plasma than in FVIII-deficient plasma with and without emicizumab, and it depended on the thrombin concentration, and the differences in the CWA-TT between normal and FVIII-deficient plasma with and without emicizumab were significant (Figure 3).

Regarding mixing tests with normal and FVIII-deficient plasma, the CWA-APTT showed large differences between mixing plasma with and without emicizumab (0.15 mg/mL), although the peak times were gradually shortened, and the peak heights showed a gradual, concentration-dependent increase in normal plasma (Supplementary Figure S3a). In contrast, the CWA-TT showed a similar pattern between mixing plasma with and without emicizumab (0.15 mg/mL), and the second peak time of the 1st DP was gradually shortened, and the second peak height of the 1st DP and peak height of the FF curve showed a gradual, concentration-dependent increase in normal plasma (Supplementary Figure S3b).

**Figure 4:**
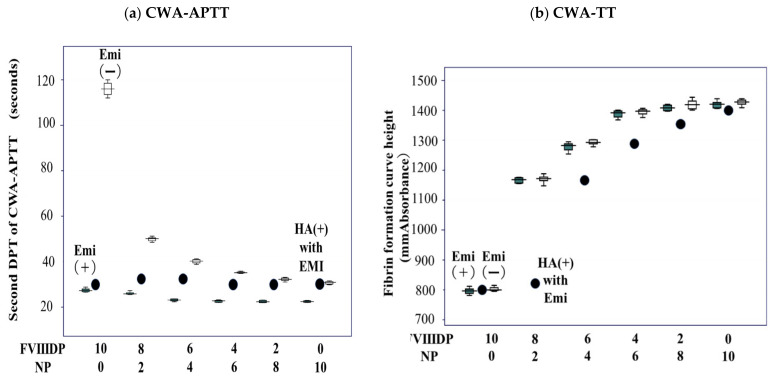
Mixing test of normal and FVIII-deficient plasma with and without emicizumab using the clot wave analysis (CWA) of the second DPT of activated partial thromboplastin time (APTT) (**a**) or fibrin formation curve height at 500 s of thrombin time (TT) (**b**). DPT, derivative peak time; FVIIIDP, FVIII-deficient plasma; NP, normal plasma; Emi (+), with emicizumab; Emi (−), without emicizumab. HA (+) with EMI (●), hemophilia A patient with inhibitor treated with emicizumab. All assays were performed more than three times to confirm the reproducibility.

The 2nd DPT of the CWA-APTT (routine APTT) showed a convex decrease in the mixing test with normal and FVIII-deficient plasma and a straight line in the mixing test with normal and FVIII-deficient plasma containing emicizumab and with plasma from a hemophilia A patient with an inhibitor treated with emicizumab and FVIII-deficient plasma (Figure 4a). The FF curve height of the CWA-TT in the mixing test with normal and FVIII-deficient plasma was convex on top, independent of the presence of emicizumab, whereas that with plasma from a hemophilia A patient with an inhibitor treated with emicizumab and FVIII-deficient plasma showed a convex, decreasing pattern (Figure 4b).

**Figure 5:**
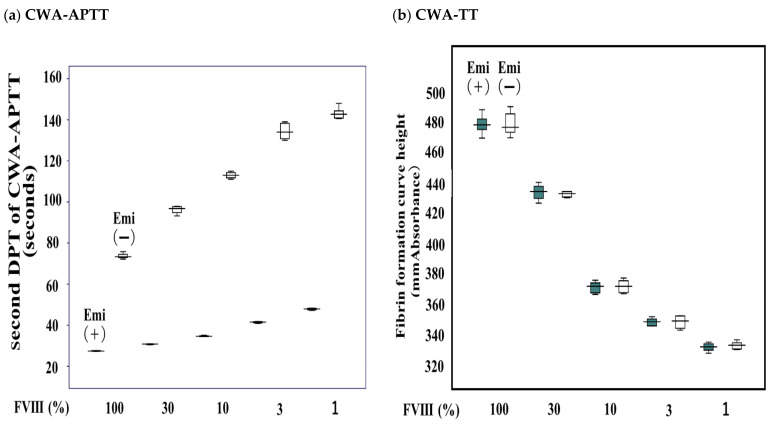
Standard curve of FVIII activity using the clot wave analysis (CWA) of activated partial thromboplastin time (APTT) (**a**) or thrombin time (TT) (**b**). Emi (+), with emicizumab; Emi (−), without emicizumab. All assays were performed more than three times to confirm the reproducibility.

**Figure 6:**
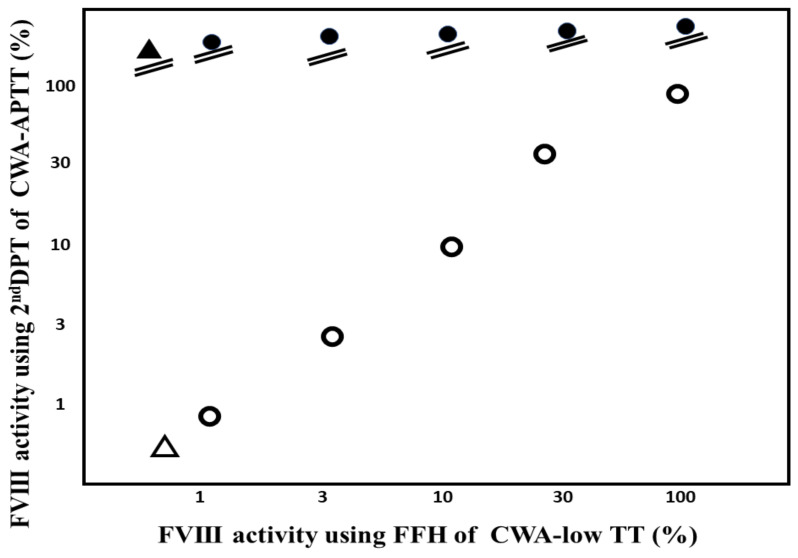
FVIII activity using the 2nd DPT of CWA-APTT or CWA low TT. CWA, clot wave analysis; APTT, activated partial thromboplastin time; low TT, low concentration of thrombin time; ●, various concentrations of FVIII plasma with emicizumab; ○, various concentrations of FVIII plasma with emicizumab; ▲, hemophilia A patient with inhibitor treated with emicizumab; △, hemophilia A patient without inhibitor treated without emicizumab.

Regarding the standard curve of FVIII activity, the 2nd DPT of CWA-APTT showed an FVIII-concentration-dependent increase, but these values with each concentration of FVIII differed between samples with and without emicizumab (Table 2) (Figure 5a), whereas the FFH of CWA-TT showed an FVIII-concentration-dependent increase, and these values for each concentration of FVIII were similar between samples with and without emicizumab (Figure 5b). The FVIII activities in various concentrations of FVIII plasma without emicizumab or plasma from a hemophilia A patient treated without emicizumab were similar between FVIII assays using the 2nd DPT of the CWA-APTT or peak heights of CWA with low TT. In contrast, all concentrations of FVIII plasma with emicizumab or plasma from a hemophilia A patient with an inhibitor treated with emicizumab showed an activity level ≥100% (Figure 6).

## 4. Discussion

Four clinical trials for emicizumab (HAVEN 1–4) in hemophilia A patients with or without inhibitors showed the significant improvement of treatments for hemophilia [6,7,24,25]. Although HAVEN 1 showed several thrombotic complications in combination with activated plasma prothrombin complex reagents [6], there were further new thrombotic complications in the long-term outcomes in the HAVEN 1–4 studies [26]. Furthermore, emicizumab will be used for patients with acquired hemophilia A. In the future, correctly measuring the FVIII activity in patients receiving emicizumab will be important for deciding whether or not to discontinue the administration of emicizumab.

A CWA is useful for evaluating not only peak time but also the peak height [18]. The peak height is useful for evaluating physiological coagulation [18,19]. The CWA-APTT is an especially useful assay for the evaluation of the effects of emicizumab in FVIII-deficient plasma; 0.00015 mg/mL of emicizumab caused a small peak of clot formation, and 0.150 mg/mL of emicizumab caused a similar peak of clot formation in normal plasma. The trough value of emicizumab was reported to be approximately 0.050 mg/mL [7], suggesting that the peak CWA-APTT may be similar to the peak in normal plasma at the peak value of emicizumab. There were no significant differences in the peak heights of CWA-APTT in normal plasma among various concentrations of emicizumab. Therefore, the risk of hypercoagulability may be low in patients receiving emicizumab monotherapy.

There were no significant differences in CWA-TT with a low concentration of thrombin between normal and FVIII-deficient plasma samples among the various concentrations of emicizumab. In contrast, the peak heights of the CWA-TT in normal plasma were significantly higher than those in FVIII-deficient plasma at each concentration of emicizumab, suggesting that the CWA-TT may reflect FVIII activity independent of the presence of emicizumab. The CWA-TT using a low concentration of thrombin (CWA–low TT) was reported to evaluate the thrombin burst phenomenon [17,27]. The mixing test of the CWA-TT using a low concentration of thrombin showed results independent of the presence of emicizumab, suggesting that this mixing test using CWA-TT is useful for detecting inhibitors in patients treated with emicizumab. In CWA–low TT, the FVIII activity can be measured using thrombin burst. A previous report [17] showed that 0.5 IU thrombin was the most useful concentration for evaluating the thrombin burst, as <0.1 IU thrombin cannot generate a significant peak, and ≥1 IU thrombin creates a fibrinogen-dependent peak with a low effect of thrombin burst. In the present study, the peak time of CWA–low TT was longer and the peak height higher with a high concentration of FVIII than with a low concentration, although the peak time of CWA-APTT was shorter and the peak height higher with a high concentration of FVIII than with a low concentration. Therefore, as the peak height of CWA–low TT showed a similar behavior to the peak height of CWA-APTT, the peak height of CWA–low TT was used in this assay.

Regarding the standard curve of FVIII activity, both the CWA-APTT and CWA-TT showed the curve of concentration-dependent FVIII activity. However, the 2nd DPT of CWA-APTT was significantly shorter in samples with emicizumab than in those without emicizumab, indicating that FVIII activities in patients treated with emicizumab were significantly higher in measurements of CWA-APTT than true FVIII activity. In contrast, the peak height of the CWA-TT showed a similar standard curve for FVIII activity between samples with and without emicizumab. Therefore, we can determine the FVIII activity of patients treated with emicizumab by CWA-TT using the standard curve of FVIII activity in normal plasma. The measurement of FVIII activity using the sTF/FIXa assay was previously reported [19]. The measurement of FVIII activity by CWA-TT may also aid in understanding the coagulation system. CWA-TT reflects thrombin burst, which depends mainly on the activation of FVIII [17]. Therefore, FVIIIa with FIXa and phospholipids activates FX without the effects of emicizumab.

**Figure 7:**
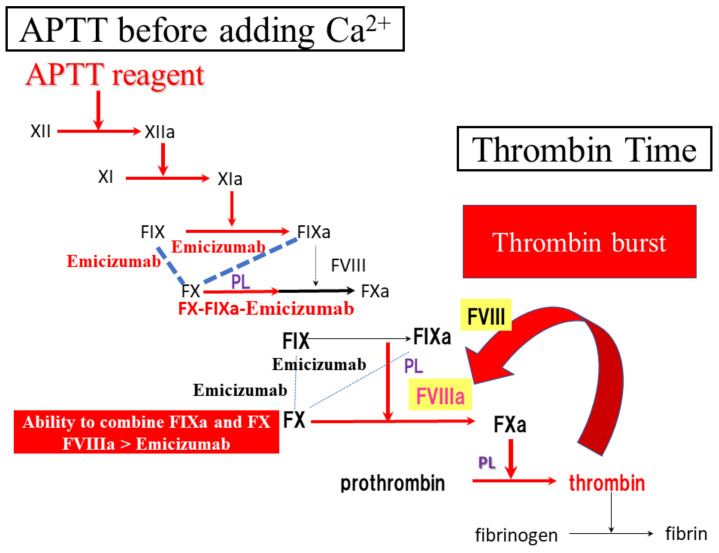
Difference between APTT and thrombin time. APTT, activated partial thromboplastin time.

Regarding the results of APTT assay, APTT reagents activate the intrinsic pathway, resulting in the generation of FIXa and FIXa and FX bound by emicizumab before addition of Ca^2+^. Therefore, a FIXa, FX and emicizumab complex without FVIII generates FXa after the addition of Ca^2+^ (Figure 7).

Regarding the limitations, the clotting assay usually depends on the fibrinogen concentration, so the findings of the clotting assay may be affected by the concentration. However, the purpose of this study was to evaluate the clotting activity independent of the presence of emicizumab. Subjects in a future study will be hemophilic patients treated with emicizumab, and their fibrinogen levels are usually within the normal range. Using a FVIII activity assay and mixing test has less dependent fibrinogen levels.

## 5. Conclusions

The CWA-TT using a small amount of thrombin can measure FVIII activity without the effects of emicizumab.

## Figures and Tables

**Table 1:** The heights of the fibrin formation curve in the clot wave analysis of thrombin time.

	FVIII-Deficient Plasma		Normal Plasma	
Thrombin Concentration	With Emicizumab	Without Emicizumab	With Emicizumab	Without Emicizumab
	(a) (mm Absorbance)	(b) (mm Absorbance)	(c) (mm Absorbance)	(d) (mm Absorbance)
1.0 IU/mL	993 ± 31 ***###	957 ± 49 ***###	1043 ± 38	1047 ± 45
0.5 IU/mL	893 ± 21 ***###	776 ± 71 ***###	1097 ± 25	1203 ± 35
0.1 IU/mL	812 ± 39 ***###	502 ± 35 ***###	1156 ± 49	1203 ± 49

The heights of fibrin formation at 400 s are shown. ***, *p* < 0.001 in comparison to (c); ###, *p* < 0.001 in comparison to (d); there were no significant differences between (a) and (b) or between (c) and (d). All assays were performed more than three times to confirm the reproducibility.

**Table 2:** FVIII activity and CWA-TT.

FVIII Activity	FFH of CWA-TT (mm Absorbance)		FFT of CWA-TT (Second)		1st DPH of CWA-TT (mm Absorbance)		1st DPT of CWA-TT (Second)	
	Emi (+)	Emi (−)	Emi (+)	Emi (−)	Emi (+)	Emi (−)	Emi (+)	Emi (−)
100%	481 ± 6.88	434 ± 8.30	186 ± 3.37	184 ± 1.56	52.7 ± 1.47	53.7 ± 1.56	33.3 ± 1.26	32.5 ± 1.52
30%	435 ± 5.46	433 ± 1.96	131 ± 6.57	127 ± 6.44	50.6 ± 2.85	49.4 ± 3.74	37.8 ± 2.05	35.4 ± 6.30
10%	370 ± 4.00	371 ± 4.55	93.3 ± 4.47	90.6 ± 2.43	50.8 ± 1.34	49.3 ± 2.29	33.8 ± 0.91	33.6 ± 1.22
3%	347 ± 2.71	347 ± 4.46	80.1 ± 3.02	81.2 ± 3.35	46.3 ± 3.82	47.2 ± 4.63	31.1 ± 2.42	30.7 ± 1.36
1%	330 ± 2.91	331 ± 2.69	73.6 ± 2.04	74.8 ± 5.25	44.8 ± 2.86	46.4 ± 3.62	33.4 ± 1.30	33.5 ± 0.86

Data are shown as the mean ± standard deviation. FFH, fibrin formation curve height; FFT, fibrin formation curve time; 1st DPH, first derivative peak height; 1st DPT, first derivative peak time; Emi (+), with emicizumab; Emi (−), without emicizumab. All assays were performed more than three times to confirm the reproducibility.

## Data Availability

The data presented in this study are available on request to the corresponding author. The data are not publicly available due to privacy restrictions.

## Supplementary Materials

The following supporting information can be downloaded at: https://www.mdpi.com/article/10.3390/jcm11206142/s1, Figure S1: Effects of emicizumab (0.15 mg/mL) on the clot waveform analysis (CWA)-small amount of tissue factor-induced FIX activation assay (sTF/FIXa); Figure S2: Effects of emicizumab on the clot waveform analysis (CWA)—thrombin time (TT); Figure S3: Effects of emicizumab on mixing test using the clot waveform analysis (CWA)-activated partial thromboplastin time (APTT) (a) and CWA-thrombin time (TT) (b). Upper, Emi (+); Lower, Emi (−) NP, normal plasma; FVIIID, FVIII-deficient plasma; Emi (+), with emicizumab; Emi (−), without emicizumab; navy blue, fibrin formation curve; pink curve, 1st derivative curve; light blue curve, 2nd derivative curve.
