# Supplementary material for: A Clot Waveform Analysis of Thrombin Time Using a Small Amount of Thrombin Is Useful for Evaluating the Clotting Activity of Plasma Independent of the Presence of Emicizumab
Source: J Clin Med. 2022 Oct 18;11(20):6142. doi: 10.3390/jcm11206142 (PMC9605059; doi:10.3390/jcm11206142)
Supplement: Supplementary file 1 [file jcm-11-06142-s001.zip › jcm-1946552-supplementary.pdf]

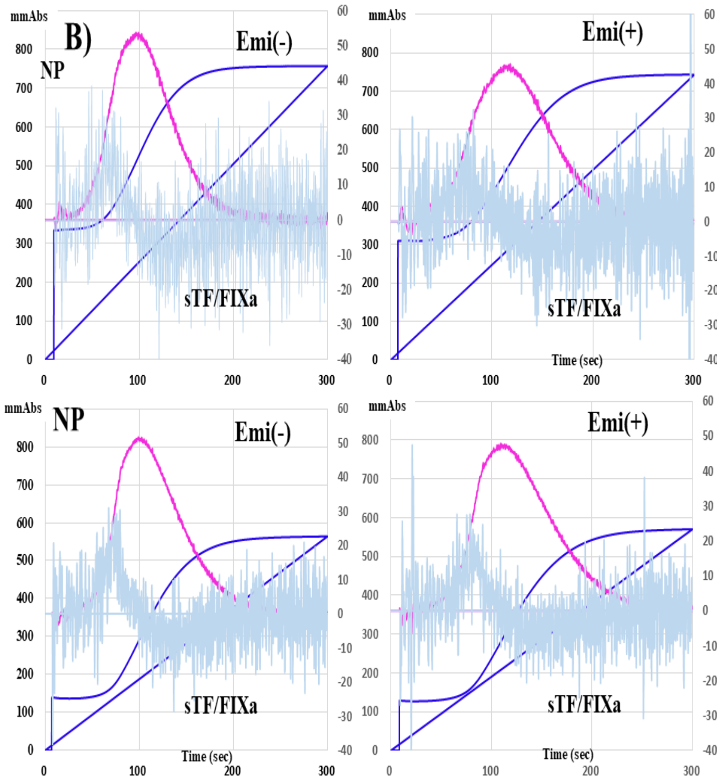

Supplementary Figure S1. Effects of emicizumab (0.15 mg/ml) on the clot waveform analysis (CWA)-small amount of tissue factor-induced FIX activation assay (sTF/FIXa)

Upper, NP (normal plasma); lower FVIIID (FVIII-deficient plasma) Emi, emicizumab; navy blue, fibrin formation curve; pink curve, 1st derivative curve; light blue curve, 2nd derivative curve.

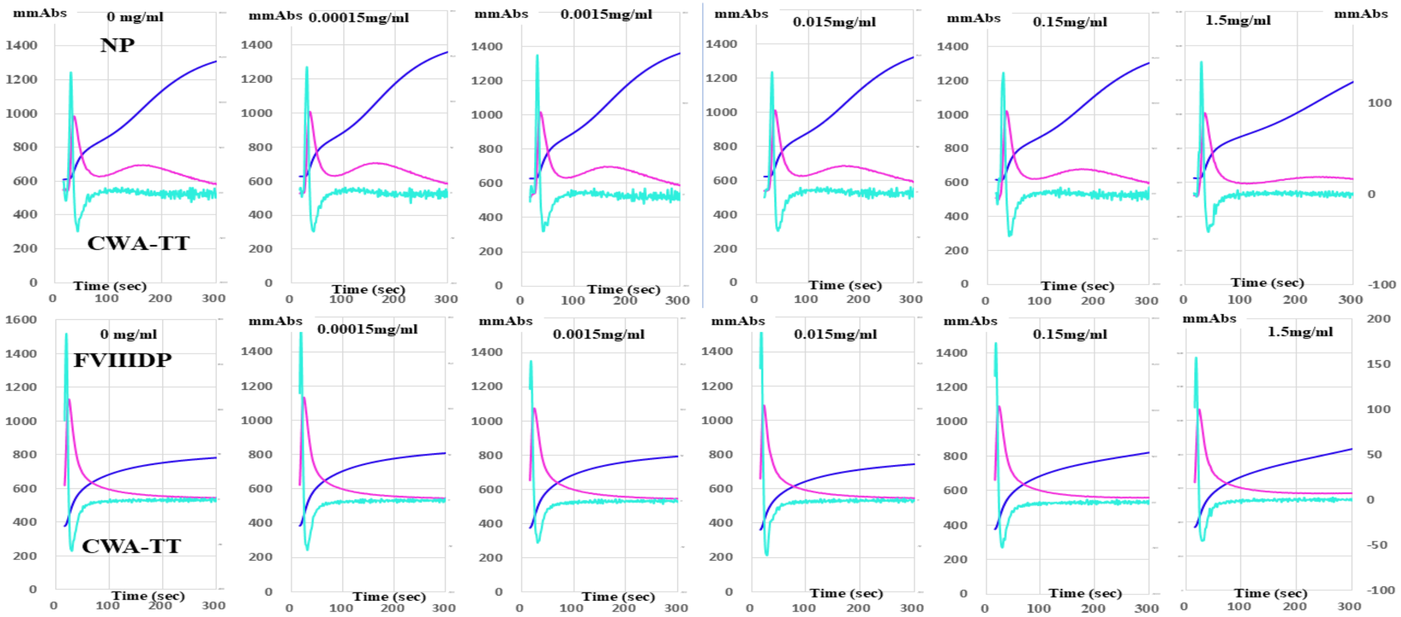

Supplementary Figure S2. Effects of emicizumab on the clot waveform analysis (CWA) - thrombin time (TT).

Upper, NP (normal plasma); FVIIID, FVIII-deficient plasma; number mg/ml,

emicizumab concentration; navy blue, fibrin formation curve; pink curve, 1[st]derivative curve; light blue curve, 2[nd]derivative curve.

a) CWA-APTT

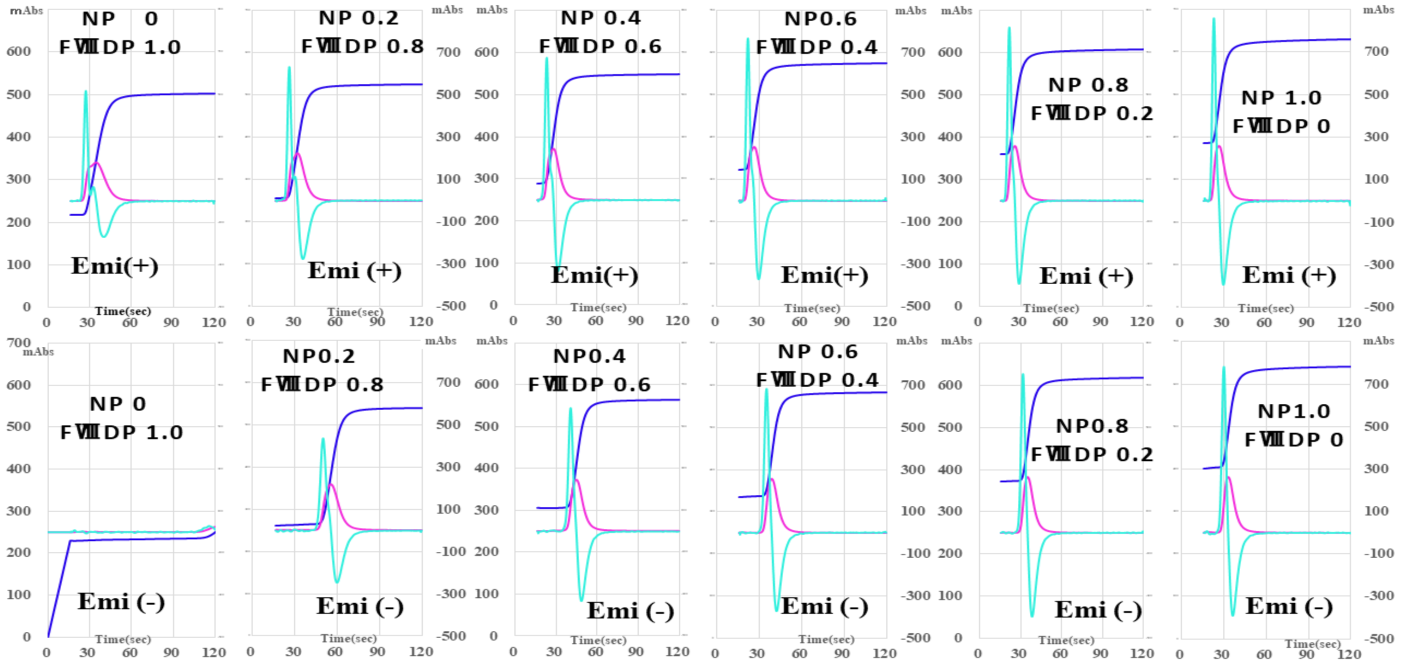

NP 0
FⅧDP 1.0
Emi(+)
NP 0.2
FⅧDP 0.8
Emi (+)
NP 0.4
FⅧDP 0.6
Emi(+)
NP0.6
FⅧDP 0.4
Emi(+)
NP 0.8
FⅧDP 0.2
Emi (+)
NP 1.0
FⅧDP 0
Emi (+)
NP 0
FⅧDP 1.0
Emi (-)
NP0.2
FⅧDP 0.8
Emi (-)
NP0.4
FⅧDP 0.6
Emi (-)
NP 0.6
FⅧDP 0.4
Emi (-)
NP0.8
FⅧDP 0.2
Emi (-)
NP1.0
FⅧDP 0
Emi (-)
mAbs
Time(sec)

b) CWA-APTT

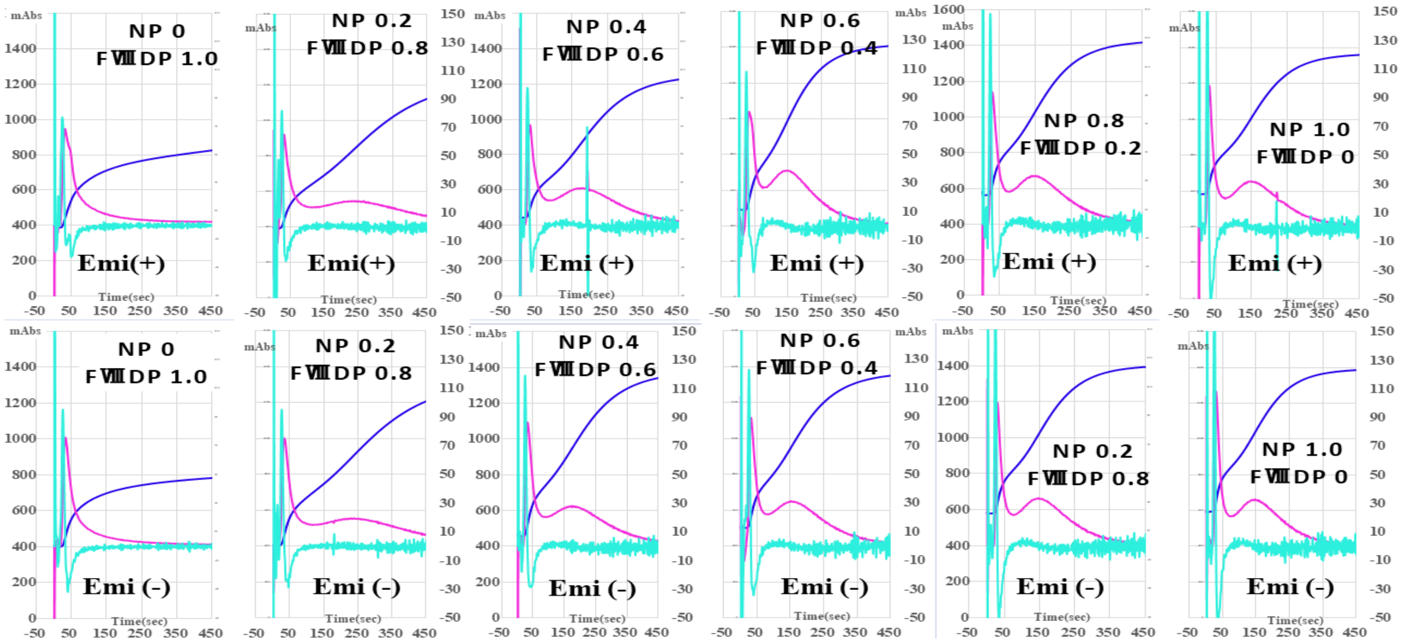

Supplementary Figure S3. Effects of emicizumab on mixing test using the clot waveform analysis (CWA) -activated partial thromboplastin time (APTT) (a) and CWA-thrombin time (TT) (b). Upper, Emi (+); Lower, Emi (-) NP, normal plasma; FVIIID, FVIII-deficient plasma; Emi (+), with emicizumab; Emi (-), without emicizumab; navy blue, fibrin formation curve; pink curve, 1st derivative curve; light blue curve, 2ndderivative curve.
